# Olivocochlear Efferents in Animals and Humans: From Anatomy to Clinical Relevance

**DOI:** 10.3389/fneur.2018.00197

**Published:** 2018-03-26

**Authors:** Enrique A. Lopez-Poveda

**Affiliations:** ^1^Instituto de Neurociencias de Castilla y León, Universidad de Salamanca, Salamanca, Spain; ^2^Departamento de Cirugía, Facultad de Medicina, Universidad de Salamanca, Salamanca, Spain; ^3^Instituto de Investigación Biomédica de Salamanca, Universidad de Salamanca, Salamanca, Spain

**Keywords:** attention, cochlear implants, olivocochlear reflex, learning, otoacoustic emissions, psychoacoustics, speech-in-noise, superior olivary complex

## Abstract

Olivocochlear efferents allow the central auditory system to adjust the functioning of the inner ear during active and passive listening. While many aspects of efferent anatomy, physiology and function are well established, others remain controversial. This article reviews the current knowledge on olivocochlear efferents, with emphasis on human medial efferents. The review covers (1) the anatomy and physiology of olivocochlear efferents in animals; (2) the methods used for investigating this auditory feedback system in humans, their limitations and best practices; (3) the characteristics of medial-olivocochlear efferents in humans, with a critical analysis of some discrepancies across human studies and between animal and human studies; (4) the possible roles of olivocochlear efferents in hearing, discussing the evidence in favor and against their role in facilitating the detection of signals in noise and in protecting the auditory system from excessive acoustic stimulation; and (5) the emerging association between abnormal olivocochlear efferent function and several health conditions. Finally, we summarize some open issues and introduce promising approaches for investigating the roles of efferents in human hearing using cochlear implants.

## Introduction

The auditory nervous system is continuously sensing and interpreting the sounds around us. Our ears operate as the sound detectors, transducing acoustic pressure into auditory nerve action potentials, and coding the characteristics of sounds appropriately for further processing by the central auditory system. The ears, however, do not work as fixed sound receptors. Instead, the central nervous system can adjust their functioning, and thus the coding of sounds, via olivocochlear efferents. Olivocochlear efferents can be activated by selective attention and/or by sounds presented to either or both ears. Therefore, the functioning of the ears is changing dynamically over time, during natural active and passive listening.

While many aspects of efferent anatomy, physiology, and function are well established, others remain controversial. This article reviews the current knowledge on olivocochlear efferents, with emphasis on human medial efferents. In Section “Anatomy and Physiology of Olivocochlear Efferents in Animals,” we review the basic anatomical and physiological characteristics of olivocochlear efferents in animals, highlighting new findings. In Section “Olivocochlear Efferent Effects in Humans,” we review the methods typically used for investigating the medial-olivocochlear efferents in humans [vestibular neurectomy, otoacoustic emissions (OAEs), and psychoacoustics], we discuss the limitations of each method and provide some good-practice recommendations. Section “Olivocochlear Efferent Effects in Humans” is also devoted to reviewing the characteristics of medial-olivocochlear efferents in humans. In Section “Roles of the Olivocochlear Efferent Reflexes in Human Hearing,” we review the possible roles of olivocochlear efferents in hearing, including their role in facilitating a normal development of the auditory system, in protecting the auditory system from acoustic overstimulation, and in facilitating the detection and recognition of signals embedded in noise. Section “Clinical Relevance and Special Populations” provides a brief review of the emerging association between abnormal olivocochlear efferent function and several health conditions. Finally, Section “Open Issues and Outlook” describes open issues and new promising approaches for investigating the roles of olivocochlear efferents in human hearing using cochlear implants.

The review spans from the early to the most recent studies. Although comprehensive, however, the cited literature is limited and possibly biased. The interested reader may broaden his/her scope by reading other excellent reviews on this topic (1–8).

## Anatomy and Physiology of Olivocochlear Efferents in Animals

### Anatomy of Olivocochlear Efferents

**Figure 1:**
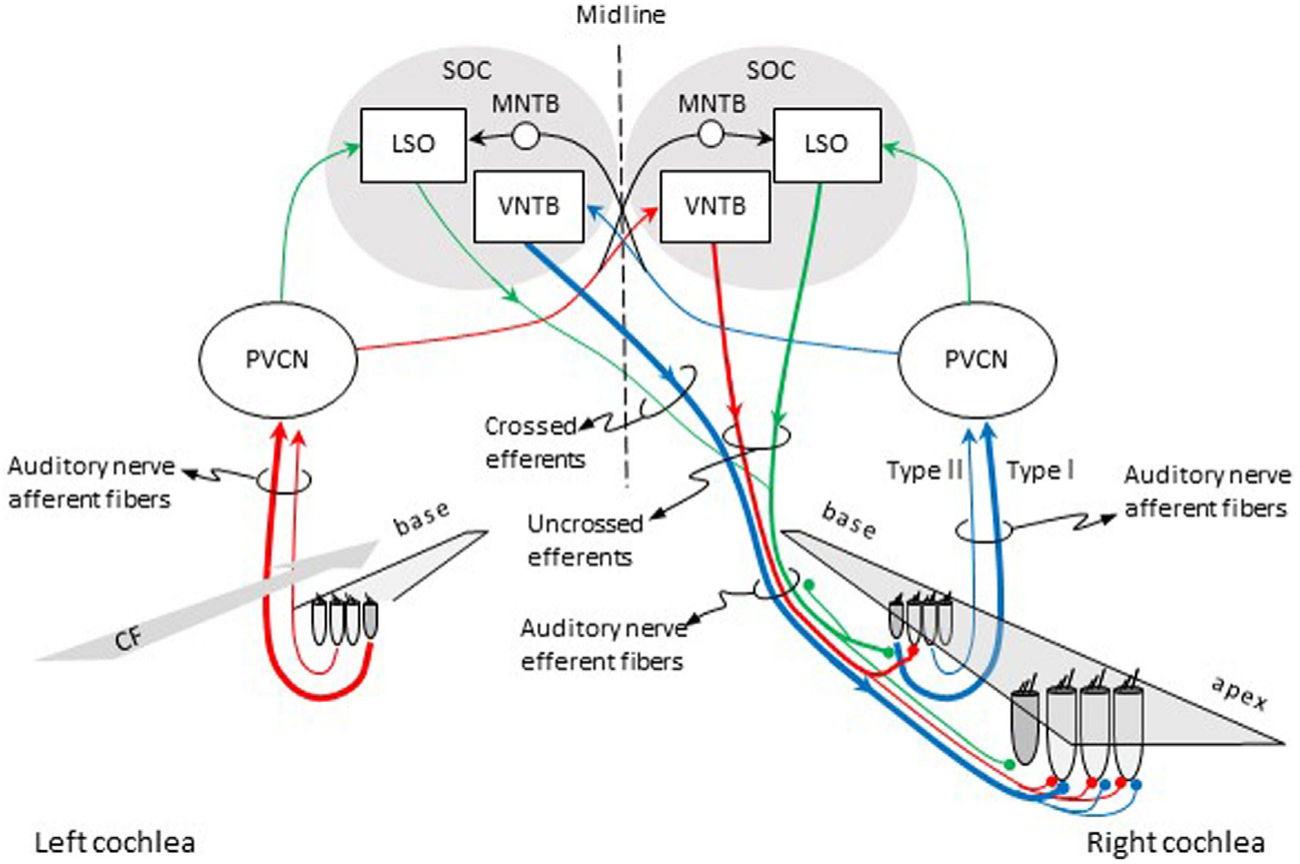
Pathways for activation of medial (MOC) and lateral olivocochlear (LOC) efferent fibers to the right cochlea. Red and blue lines illustrate the pathways for activation of the contralateral and ipsilateral MOC reflexes, respectively. Green lines illustrate the pathways for activation of the LOC reflex. The thickness of each line roughly illustrates the density of innervation. Abbreviations: LSO, lateral superior olive; MNTB, medial nucleus of the trapezoid body; PVCN, postero-ventral cochlear nucleus; SOC, superior olivary complex; VNTB, ventral nucleus of the trapezoid body; CF, characteristic frequency.

Olivocochlear efferent fibers originate in the left and right superior olivary complexes (SOCs), project to the cochlea through the vestibular nerve, enter the basal turn of the cochlea along with auditory nerve afferent fibers, and terminate in the organ of Corti. They were first described by Rasmussen (9), who originally classified them into crossed and uncrossed types, depending on whether they originated in the contralateral or the ipsilateral SOC, respectively. At present, efferents are classified into medial and lateral based upon the location of their parent cells bodies in the SOC and their site of termination (10, 11). Medial-olivocochlear (MOC) efferents originate in the medial superior olivary nuclei and terminate directly upon outer hair cells (OHCs), while lateral olivocochlear (LOC) efferents originate in the lateral superior olivary nuclei and terminate on the dendrites of type I auditory nerve afferent fibers, beneath inner hair cells (IHCs) (Figure 1).

In cat, there are about 850 LOC and 500 MOC fibers (12). In human, there are on average 1005 LOC fibers and 360 MOC fibers, although the actual number can vary across individuals (13). Both LOC and MOC efferents contain crossed (contralateral) and uncrossed (ipsilateral) fibers. In most mammals, however, the majority of LOC fibers project to the ipsilateral cochlea (14) while the majority of MOC fibers project to the contralateral cochlea (15). The density of efferent innervation in the cochlea varies across species. In cat, a greater number of MOC fibers terminate near the center of the cochlea [i.e., at cochlear sites with characteristic frequencies (CFs) between 1 and 10 kHz] than at the cochlear ends. Crossed MOC fibers are more numerous toward the cochlear base, and uncrossed MOC fibers are more evenly distributed over the 1–10 kHz CF range. Crossed LOC fibers are scarce but relatively more numerous in the cochlear apex while uncrossed LOC fibers are more numerous and evenly distributed throughout the cochlea [see Fig. 8.3 in Ref. (2)].

In cat, each MOC efferent fiber can make contact with 23–84 OHCs, spanning 3.2 mm along the cochlear length, which corresponds to roughly an octave of afferent CF (16). In guinea pig, each MOC efferent fiber can make contact with between 14 and 69 OHCs, can span up 24% of the total cochlear length (nearly two octaves in sound frequency), and the number of contacts decreases with increasing CF (17). It is commonly assumed that the innervation for MOC efferents that respond to ipsilateral (crossed efferents) and contralateral sounds (uncrossed efferents) is similar. However, in guinea pig, efferents that respond to contralateral sounds (uncrossed efferents) innervate a cochlear region twice as large as efferents that respond to ipsilateral (crossed efferent) sounds. This suggests differences in the functional roles for the two types of MOC neurons (17). MOC efferent fibers terminate on OHCs corresponding with cochlear regions with CFs equal or lower to the CFs of the auditory nerve afferents (as illustrated by the red lines in Figure 1).

In rat, LOC neurons have been classified in two types: small neurons confined to the LSO (called “intrinsic” neurons) and large neurons that closely surround the LSO (called “shell” neurons) (18). While the projections from the two types of LOC neurons terminate beneath the IHCs, the pattern of terminations is different for each one of them. The axons of intrinsic neurons do not bifurcate upon entering the cochlea and terminate in dense patches spanning 10–20% of the total length of the organ of Corti. By contrast, the axons of “shell” neurons bifurcate upon entering the cochlea into apical and basal branches and their terminations span more than 50% of the cochlear length (19).

The anatomy of olivocochlear neurons and their projections to the cochlea varies across species. A detailed comparison of differences across species is out of the scope of this review. The interested reader is referred to the review of Warr (20), and in particular to his Table 7.1.

### Efferent Neurotransmitters

Acetylcholine is the major neurotransmitter of MOC and LOC efferents (i.e., most efferent fibers are cholinergic), although there is evidence indicating co-localization of calcitonin gene-related peptide, and γ-aminobutyric acid [reviewed in Ref. (1, 21)]. In addition, a small subgroup of LOC neurons is dopaminergic (22).

### Efferents Response to Sound

#### Pathways for the Olivocochlear Reflexes

Olivocochlear efferents respond to sound, hence the term olivocochlear efferent “reflexes.” The pathways for the activation of the reflexes are illustrated in Figure 1. For the contralateral MOC reflex (red lines in Figure 1), sounds presented to the left-ear activate auditory nerve afferent fibers, which project to neurons in the postero-ventral cochlear nucleus (PVCN) (commonly referred to as MOC-reflex interneurons). MOC interneurons project to MOC neurons in the contralateral ventral nucleus of the trapezoid body (VNTB), which project to the OHCs in the cochlea in the right ear [see Ref. (23), for evidence of this pathway in guinea pigs]. For the ipsilateral MOC reflex (blue lines in Figure 1), sounds presented to the right ear activate auditory nerve afferent fibers, which project to MOC interneurons in the ipsilateral PVCN, which project to the contralateral MOC neurons in the VNTB, which project contralaterally to the right cochlea [see Ref. (24), for evidence of this pathway in mouse]. In other words, the pathway for the contralateral MOC reflex involves *uncrossed* efferent fibers and a single crossing of the brainstem midline, while the pathway for the ipsilateral MOC reflex involves *crossed* efferent fibers and a double crossing of the midline. The activity of MOC efferents is modulated by direct projections from higher centers of the auditory pathway, including the inferior colliculus and the cerebral auditory cortex [reviewed in Ref. (25, 26)].

Some studies have argued that, in guinea pig, the MOC reflexes start with activation of auditory nerve afferents that innervate the IHCs (type I afferents) [e.g., Ref. (23)]. Froud et al. (27), however, found absent MOC reflexes for a mutant mouse presumably lacking type II afferents, and thus suggested that it is type II auditory nerve afferents that initiate the MOC reflex. The latter is interesting because type II afferents, which are only 5% of the afferent population, innervate OHCs rather than IHCs, and their role in hearing has been long uncertain. In addition, it would imply that the MOC reflex starts and ends at the OHCs. Maison et al. (28), however, dispute this view on the grounds that the lack of MOC reflexes in the mutant used by Froud et al. is due to a defect in efferent transmission rather than a loss of sensory drive.

The green lines in Figure 1 depict the pathways for the LOC reflex. Auditory nerve fibers project to planar multipolar neurons in the PVCN [e.g., Ref. (29)]. Although not yet directly demonstrated, in rat, planar multipolar cells on either side of the brain probably send innervation to ipsilateral LOC neurons [the evidenced is discussed by Gómez-Álvarez and Saldaña (30)]; hence, LOC neurons can be probably activated by sound. LOC neurons then project to type I afferent fibers in the ipsilateral and contralateral cochleae. The majority of LOC efferent fibers are uncrossed. Therefore, the LOC reflex is thought to be predominantly ipsilateral [reviewed in Ref. (3)].

#### Physiological Response to Sound

It is possible to measure the response of individual efferent fibers to sounds by placing a recording electrode in the saccular ganglion. It is difficult, however, to differentiate if a measured response is from an MOC or an LOC efferent fiber. Lateral efferent fibers are thinner and unmyelinated, while MOC efferent fibers are thicker and myelinated. Because it is difficult to record responses from unmyelinated fibers, most recordings of efferent responses to sound are thought to come from MOC efferents (16).

In cat, electrophysiological recordings of single, presumably MOC, efferent fibers to sound have shown (16) that efferents (1) fire regularly over time to low-level noise or tone bursts, a characteristic that helps to differentiate efferent from afferent fibers, which fire irregularly; (2) their inter-spike intervals appear correlated with sound level rather than with the period of the stimulus frequency; (3) do not respond or respond minimally to sound durations less than about 25 ms; (4) 59% of efferents respond to ipsilateral sound stimulation, 29% to contralateral sounds, and 11% respond to sounds presented to either ear; (5) for some fibers that respond to sound presented to either ear, opposite ear stimulation can decrease the saturated discharge rate to ipsilateral stimulation; (6) very few (14%) efferent fibers have spontaneous activity and most of those respond to sounds presented to either ear; (7) efferent fibers are tuned in frequency but slightly more broadly tuned on average than afferent fibers; (8) the CF of efferent fibers seem to correspond with the CF of the afferent fibers [but see Ref. (17, 31), for contradicting results in guinea pigs]; and (9) contralateral efferents can be found in all cochlear regions while either-ear efferents are more common for CFs < 2 kHz.

### Effects of Olivocochlear Efferent Activation

#### Effects in Silent Backgrounds

**Figure 2:**
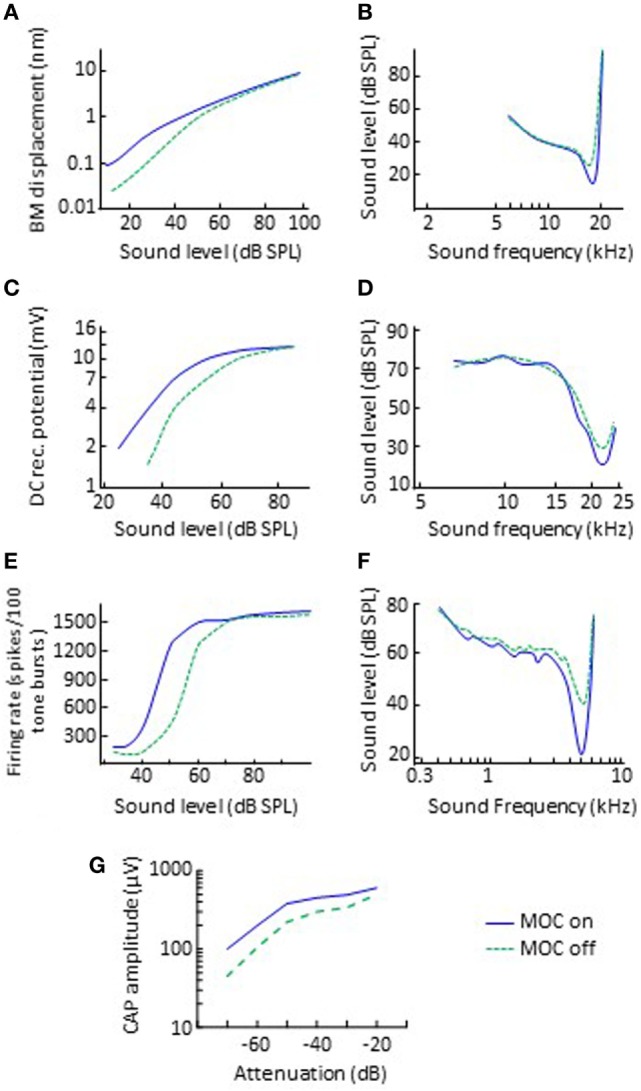
Effects of electrical activation of olivocochlear efferents on various physiological responses to sounds in quiet. The left and right panels illustrate input/output and threshold tuning curves for the corresponding system, respectively. **(A,B)** Basilar motion. Data re-plotted, in modified form, from Cooper and Guinan (33). **(C,D)** Inner hair cell receptor potential. Data re-plotted, in modified form, from Brown and Nuttall (34). **(E,F)** Discharge of single auditory nerve afferent fibers. Data in panels **(E,F)** are re-plotted, in modified form, from Wiederhold (35) and Guinan and Gifford (36), respectively. **(G)** Auditory nerve CAP. Data re-plotted, in modified form, from Elgueda et al. (37). Abbreviations: BM, basilar membrane; CAP, compound action potential; DC, direct current; MOC, medial-olivocochlear efferents.

In animal preparations, it is possible to activate olivocochlear efferents with electrical shocks while measuring cochlear physiological responses to sound. Typically, the electrical shocks are delivered with an electrode placed at the midline of the floor of the fourth ventricle and thus probably activate both the crossed and uncrossed MOC fibers but not LOC fibers [evidence reviewed in p. 445 of Ref. (2)]. Using this approach, researchers have found that the main effect of MOC efferent activation is to inhibit (reduce) the amplitude of mechanical vibration of the organ of Corti in response to sounds of low-to-moderate intensity and with frequencies close to the CF of the recording site in the cochlea (32, 33). In other words, MOC efferent activation linearizes cochlear mechanical input–output curves (Figure 2A) and broadens the corresponding threshold tuning curves by shifting their tips upwards (Figure 2B).

The presumed mechanism is as follows. The organ of Corti vibrates in response to sound. This vibration can increase (depolarize) or decrease (hyperpolarize) the membrane voltage of IHCs and OHCs located in the cochlear region tuned to the sound frequency (depolarization can eventually cause type I and type II auditory nerve afferent fibers to fire, and thus signal sounds to the auditory brain). The electrical changes in the OHC membrane potential can cause OHCs to contract and expand, a property termed electromotility (38, 39). The amplitude of vibration of the organ of Corti is larger with normal OHC electromotility than without it, indicating that the OHCs are crucial for enhancing the mechanical vibration of the organ of Corti in response to sounds of low-to-moderate intensity (40). Medial efferents terminate directly on the basolateral membrane of OHCs and their synapses are cholinergic. Activation of MOC efferents hyperpolarizes the OHCs, presumably inhibiting OHC electromotility (41, 42), and thereby inhibiting the amplification effect of OHCs and reducing the amplitude of vibration of the organ of Corti (32, 33).

Because the motion of the organ of Corti triggers the chain of events involved in hearing, the inhibitory effect MOC efferent activation on the mechanical motion of the organ of Corti reflects on multiple other cochlear physiological responses to sound. Interestingly, some of the effects on these other measurements were observed long before the effect of MOC efferent activation on the vibration of the organ of Corti. Among these other effects are the following:

1. A small *increase* in the cochlear microphonic (37, 43), a cochlear voltage that is dominated by the OHCs and mirrors the waveform of the acoustic stimulus. Note that electro-cochleography is a clinical technique for measuring the cochlear microphonic.
2. A reduction of up to 10 mV in the endocochlear potential (44, 45), the driving “battery” for mechano-electrical transduction in IHC.
3. A reduction in the amplitude of the alternate- and direct-current components of the IHC receptor potentials (Figure 2C), accompanied by an increase in threshold and broadening at the tips of the frequency tuning curves of IHCs (Figure 2D) (34, 46).
4. A reduction in the discharge rate of individual auditory nerve fibers. The effect is to horizontally shift the dynamic range of individual auditory nerve fibers to higher levels by up 25 dB (or 10 dB in the example shown in Figure 2E) (35), accompanied by an increase in threshold and broadening at the tips of the fibers’ frequency tuning curves (Figure 2F) (36).
5. A reduction in the amplitude of the auditory nerve compound action potential (CAP) to low-level but not high-level clicks (Figure 2G) (37, 47). The effect is to horizontally shift the low-level portion of the CAP versus level function by up 18 dB. Note that the CAP is equivalent to the clinical wave-I auditory evoked potential.
6. A change in the levels of OAEs (48, 49). OAEs are sounds generated by the non-linear the vibration of the organ of Corti that can propagate “backward” through the middle ear to the auditory canal where they can be recorded using sensitive equipment (50). The activation of olivocochlear efferents linearizes cochlear mechanical responses, which in turn can increase or decrease OAE levels, as reviewed in the following sections.

#### Effects in Noisy Backgrounds

**Figure 3:**
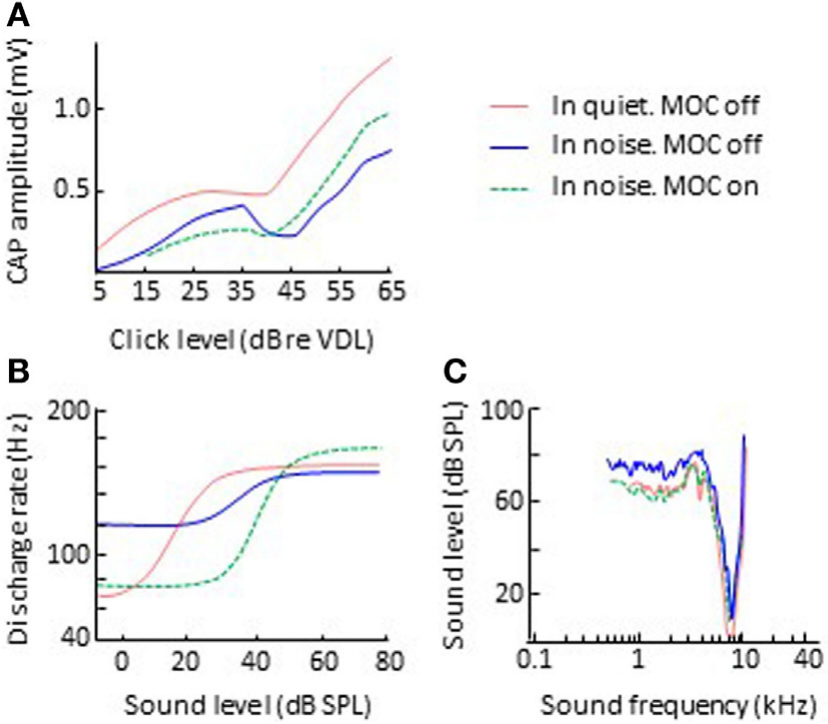
Effects of olivocochlear efferent activation on physiological responses to sound in noise. The left and right panels illustrate corresponding input/output and threshold tuning curves, respectively. **(B,C)** Single auditory nerve fiber responses. Data in panels **(B,C)** are re-plotted, in modified form, from Winslow and Sachs (54) and Kawase et al. (55), respectively. **(A)** CAP. Data in panel A re-plotted, in modified form, from Nieder and Nieder (52). Abbreviations: CAP, compound action potential; VDL, visual detection level.

While the electrical activation of olivocochlear efferents generally suppresses (inhibits) cochlear responses to sounds in silent backgrounds, it can *enhance* cochlear responses to transient stimuli in noisy backgrounds. Nieder and Nieder (51, 52) observed that the electrical activation of the olivocochlear efferents always decreased the magnitude of CAP to clicks in silent backgrounds (Figure 3A). In noise backgrounds, however, the CAP response to high-level clicks was larger with activation of olivocochlear efferents than without it. They referred to this phenomenon as an “antimasking effect.” The antimasking effect on CAP responses also occurs for brief tone bursts with moderate level sounds (50–80 dB SPL) and is greater for signal-to-noise ratios less than 20 dB (53).

Related antimasking effects have been observed in the discharge rate of single afferent auditory nerve fibers. Winslow and Sachs (56) measured the discharge rate of individual auditory nerve fibers for 200-ms pure tones in noise as a function of tone intensity, with and without electrical activation of olivocochlear efferents. Without efferent activation, the rate-intensity function for tones in noise was more “compressed” than for tones in quiet backgrounds (Figure 3B). This is presumably because for low-intensity tones, the fiber is responding to the background noise and for high-intensity tones, the fiber’s discharge rate is adapted to the noise and an adapted auditory nerve fiber is less responsive than an unadapted one (57, 58). The electrical activation of olivocochlear efferents had two effects: (1) to shift the rate-intensity functions horizontally to the higher sound levels, a result consistent with overall inhibitory effect of olivocochlear efferent activation; and (2) to “decompress” the rate-intensity functions (compare the green-dashed and blue lines in Figure 3B), thus restoring the dynamic range of auditory nerve fibers in noise to the values observed in quiet. The latter was consistent with the antimasking effect of olivocochlear efferent activation and was the result of olivocochlear efferents inhibiting the cochlear mechanical response to the noise + tone stimulus. For low-intensity tones, this had the effect of reducing the response to the noise background and for moderate-intensity tones it had the effect of reducing the adaptation to the noise.

In a subsequent study, Winslow and Sachs (54) showed that the restoration of the dynamic range of auditory nerve afferent fibers can facilitate the coding and detection of changes in intensity in noisy backgrounds, giving rise to the notion that olivocochlear efferents facilitate hearing in noise. The evidence in support and against this notion is reviewed in a later section.

Although the effects described in this section and in the previous section were observed by stimulating olivocochlear efferents with electrical shocks, the majority of those effects have been also observed by stimulating MOC efferents with contralateral sound [e.g., Ref. (55, 59–62)]. The effects have also been corroborated by comparing cochlear responses before and after cutting the olivocochlear bundle (55, 62) and with computational models of the peripheral auditory system (63, 64). This has given rise to the notion that the effects of olivocochlear efferent activation and the roles of olivocochlear efferents in hearing may be investigated by activating efferents with contralateral sounds and/or using computational models.

The “antimasking” effect of MOC efferent activation by contralateral noise occurs even when the contralateral noise is uncorrelated with the ipsilateral masking noise [Fig. 4 in Ref. (62)]. Therefore, this “antimasking” effect is different from binaural unmasking, which by definition occurs when a binaural masker is correlated and the signal is monaural or binaurally uncorrelated [e.g., Ref. (65)]. In addition, activation of the contralateral MOC reflex by contralateral noise can enhance cochlear responses even in the absence of a masker, perhaps because MOC efferent activation unmasks auditory nerve responses from own noise [e.g., Fig. 7 in Ref. (62)].

#### Time Course of Olivocochlear Efferent Effects

The inhibitory effect of olivocochlear activation on sound-evoked responses is not instantaneous. Wiederhold and Kiang (66) observed that the suppressive effect of olivocochlear efferent activation with trains of electrical shocks on the discharge rate of individual auditory nerve fibers (1) built up to its maximum level within 100 ms after shock-train onset, (2) could be maintained for many minutes with continued efferent stimulation, and (3) dissipated exponentially over 100 ms after shock-train offset. Warren and Liberman (61) reported slightly longer activation time constants (of about 100–200 ms) for acoustical activation of olivocochlear efferents, perhaps because the discharge rate of efferents to contralateral sound stimulation was less (60–80 spikes/s) than the 400 pulses/s used by Wiederhold and Kiang (66). Liberman et al. (67) observed OAE levels adapting within about 130 ms of the onset of the eliciting primary tones, presumably because the primary tones used to evoke the OAEs activated the ipsilateral efferent reflex. They also observed OAE suppression by contralateral sound stimulation with an exponential time constant of about 150 ms.

Recent studies have shown, however, that inhibition of single auditory nerve responses actually develops over two times scales (68). There is a “fast” effect, with inhibition building over tens of milliseconds, and a “slow” effect, with inhibition building over tens of seconds. The two time scales have been corroborated with direct recordings of basilar membrane motion (69) and probably emanate from different underlying mechanisms in OHC electromotility (33).

In cats, the time constant for olivocochlear efferent inhibition typically increases with increasing CF and the time constant of recovery from inhibition decreases with increasing CF (66). The time constants decrease with increasing stimulus frequency (67) and with increasing the efficiency of the olivocochlear efferent elicitors (68).

#### Effects of LOC Efferent Activation

The effects described earlier are probably due solely or mostly to the activation of MOC efferents. The peripheral effects of selective LOC efferent activation are less well established because the unmyelinated axons of LOC fibers are difficult to stimulate electrically. Groff and Liberman (70) activated LOC efferents indirectly, by placing stimulating electrodes in the inferior colliculus. LOC efferent activation enhanced or suppressed the CAP (or the round window noise), depending on the place of stimulation in the inferior colliculus. In contrast with MOC effects, the effects of LOC activation were level independent and were long lasting (lasted for 5–20 min). As expected, LOC efferent activation caused minimal changes in the cochlear microphonic or in the levels of distortion product OAEs (DPOAEs), two measures related with OHC function.

The effects of LOC efferent activation have also been investigated by selectively lesioning LOC efferents or their parent neurons in the LSO. Selective lesioning of parent neurons by injecting a cytotoxic chemical in the LSO *reduces* sound-evoked CAP amplitudes in guinea pig (71) but *enhances* auditory brainstem response wave-I amplitudes in mice (72). In other words, the effect of damaging LOC parent neurons on auditory nerve responses seems to be species specific. Disruption of LOC efferents with a dopaminergic neurotoxin *depresses* the spontaneous activity of auditory nerve fibers in guinea pig (73).

### MOC Efferents and the Cochlear Amplifier

Because MOC efferents inhibit the cochlear amplifier, it is tempting to assume that their effects must be larger for cochlear regions and stimuli where the amplifier is most effective. Many experimental observations appear consistent with this assumption. For example, the suppressive effect of efferent activation in silent backgrounds is overall larger for low-intensity sounds [e.g., Ref. (35, 37, 47)] and for stimulus frequencies around the CF (36, 55). In addition, the suppressive effects of efferent activation in silent backgrounds and the antimasking effects in noise are larger for auditory nerve fibers with higher than with lower CFs (62). These characteristics are indeed broadly consistent with the gain of the cochlear amplifier being larger in the cochlear base than in the apex, and larger for sounds of low intensity and with frequencies around the CF (40).

However, the assumption that MOC efferent effects are restricted to stimuli affected by the cochlear amplifier is somewhat inconsistent with the fact that most of the MOC terminations are more apical than the OHCs involved in the cochlear amplifier for their CF (17). It is also inconsistent with the finding that MOC efferent activation can *enhance* the amplitude of vibration of the organ of Corti for sound frequencies above CF (33, 74), and with its antimasking effects in noisy backgrounds extending to the low-frequency tails in the tuning curves of auditory nerve units, as illustrated in Figure 3C (55). These findings remain to be explained.

It is also common to assume that because OAEs reflect non-linear cochlear mechanical responses, and MOC efferent activation linearizes cochlear mechanical responses, MOC efferent activation always reduces OAE levels. While generally true, that is not always the case. Olivocochlear efferent activation can *increase* the levels of DPOAEs depending on the choice of stimulus frequencies (f1 and f2) and the measured DPOAE component. The level of the 2f1 − f2 DPOAE, the component most often measured with clinical devices, typically decreases but sometimes increases with activation of MOC efferents with electrical shocks [e.g., Fig. 4 in Ref. (49)] or contralateral sounds [Fig. 7 in Ref. (67)]. By contrast, the level of the f2 − f1 DPOAE typically increases but sometimes decreases with MOC efferent activation with either electrical shocks [e.g., Fig. 4 in Ref. (49)] or contralateral sounds [Fig. 4 in Ref. (75)]. The different direction in the level change for the 2f1 − f2 and f2 − f1 DPOAE components is possibly due to MOC efferents changing the operating point of the cochlear amplifier, which can affect the 2f1 − f2 and f2 − f1 DPOAE components differently. A detailed explanation is beyond the scope of this review but may be found elsewhere [e.g., Ref. (75, 76)].

### Effects of Anesthesia and Other Drugs

The animal experiments reviewed in the preceding sections were conducted in anesthetized animals. This means that the inhibitory effect of olivocochlear efferent activation in response to sounds in silent background and the antimasking effects in noisy backgrounds remain with anesthesia [e.g., Ref. (16, 66, 67)]. Several studies have shown, however, that those effects are smaller in anesthetized than in awake animals. For example, the suppression of DPOAEs by contralateral acoustic stimulation (CAS) is stronger in awake than in the anesthetized mice (8 versus 1 dB, respectively) (77), and in awake than in anesthetized guinea pigs (5.6 versus 1.3 dB with urethane or 0.01 dB with pentobarbital) (78). Similarly, the suppression of CAP responses and the increase in cochlear microphonic produced by CAS are 1–3 dB and up to 1.9 dB larger, respectively, in awake than in anesthetized chinchilla (79). Olivocochlear efferent effects are also affected by other drugs. For example, the inhibitory effects of CAS on DPOAEs are significantly, although reversibly, reduced (indeed almost absent) after administration of gentamicin [e.g., Ref. (78, 80)]. Similarly, the suppression of the CAP and the increase in the cochlear microphonic induced by MOC stimulation with electrical shocks can be blocked reversibly with intravenous injections of strychnine (45). The potential effects of drug treatment may be important when interpreting the variability in olivocochlear efferent effects across individuals.

## Olivocochlear Efferent Effects in Humans

The former sections were devoted to reviewing the basic anatomy and physiology of olivocochlear efferents, most of which came from studies in animals. This section is devoted to reviewing the characteristics of MOC efferents effects in humans.

### Methodological Considerations

Methodological difficulties make it hard to accurately assess MOC effects in humans. First, unlike in animals, in humans it is not always possible to measure the desired response directly. For example, human cochlear mechanical responses must be *inferred* from OAEs (3), from psychoacoustical tuning curves [e.g., Ref. (81, 82)], or from behaviorally inferred cochlear input/output curves [e.g., Ref. (83, 84)], the perceptual correlates of cochlear mechanical tuning curves and input/output curves, respectively. Sometimes, the stimuli used to generate OAEs or to obtain the psychoacoustical estimates can themselves activate the MOC reflex (67, 85–87). In addition, OAEs are measured without controlling for visual attention, while psychoacoustical techniques typically involve attending to visual cues (e.g., lights presented in a computer screen). Selective attention to visual stimuli can reduce cochlear sensitivity, presumably by activation of MOC efferents (88). Because of all this, MOC effects can affect OAEs and psychoacoustical correlates of cochlear mechanical function differently to some uncertain extent, even in the absence of explicit MOC efferent elicitors.

Another complication is that MOC-induced changes in OAE levels do not always reflect the changes in cochlear mechanical motion accurately. For example, as explained earlier, DPOAE levels can decrease or increase depending on the change in the operating point of the cochlear amplifier caused by MOC efferent activation (75, 76). Also, in measuring MOC effects with the 2f1 − f2 DPOAE, the OAE level recorded in the ear canal is the vector sum of an OAE component generated at the cochlear region tuned to the frequency of the f2 primary tone (the “distortion” component) and another component reflected at the cochlear region tuned to 2f1 − f2 frequency (the “reflection” component). Each component may have its own amplitude and phase. Olivocochlear efferent activation may change the amplitude and/or the phase of the two components differently. As a result, the levels of the 2f1-f2 DPOAE may be even *enhanced* by MOC activation if MOC efferents suppress one of the component but not the other, depending on their phases [p. 454 in Ref. (2, 89)]. Because of this, Guinan et al. (85) recommended using stimulus-frequency OAEs (SFOAEs) rather than DPOAEs to assess MOC suppression. Unlike DPOAEs, SFOAEs are generated at single cochlear region (90) and thus their suppression by ipsilateral precursor sounds or CAS is thought to reflect more accurately MOC effects in that region. However, using SFOAEs is also problematic. The effect of CAS on SFOAEs can change significantly with small changes (~40 Hz) of the stimulus frequency, which renders MOC effects on an SFOAE at a single frequency a poor measure of MOC efferent strength (91). Animal and human studies have reported stronger CAS-evoked MOC effects on the f2 − f1 than on the 2f1 − f2 DPOAE [reviewed in Ref. (75)]. While this might seem to favor using the f2 − f1 rather than the 2f1 − f2 DPOAE for assessing human MOC effects, measuring the f2 − f1 DPOAE is difficult at low f2 frequencies (92).

A second difficulty in measuring human MOC effects is that in humans, unlike in animals, it is not possible to compare sound-evoked responses before and after cutting the olivocochlear bundle. Researchers have compared responses in the same ear before and after vestibular neurectomy, or between neurectomized and control ears in the same subject [e.g., Ref. (93, 94)]. This approach, however, is impractical for regular laboratory testing, and there are a limited number of neurectomized people. It is also questionable that vestibular neurectomy cuts the olivocochlear efferents (95). Even if it did, it is conceivable that the effects of cutting the olivocochlear bundle in altering auditory function may be compensated by (re)learning new cues in the time lapse between surgery and testing.

A third difficulty is that, in humans MOC efferents cannot be activated by delivering electrical shocks in the floor of the fourth ventricle. Instead, it is common to compare sound-evoked responses in one ear in the presence and in the absence of simultaneous CAS or an ipsilateral precursor sound. The assumption is made that any difference in the response obtained in the two conditions is *only* due to the CAS or the precursor activating the contralateral and ipsilateral MOC reflexes, respectively. This assumption, however, may not hold, for example, when the CAS or the precursor sound, alone or in combination with the probe sounds used to assess the MOC effect, also activates the middle-ear muscle reflex (MEMR) (96). It would also be incorrect when the CAS interacts with the sounds used to assess the MOC effect, as discussed earlier.

Some of these issues have been noticed relatively recently. Many of the early human studies were conducted disregarding the above issues, something that probably contributed to the reported disparities in the type, direction, and magnitude of the MOC effects across studies and across individuals (91).

#### “Good-Practice” Procedures

Measures can be taken to minimize or overcome the methodological issues highlighted above. For example, to minimize the potentially confounding effects of the MEMR, it would be necessary to use CAS or ipsilateral precursors with levels lower than the individual threshold of activation of the MEMR [with can be as low as 50–55 dB SPL for some listeners according to Feeny et al. (97)]. It would be even better to test that the MOC eliciting sound together with the probe stimuli used to assess the MOC effect do not activate the MEMR, although this requires more sophisticated techniques [e.g., Ref. (82, 98)].

Given the difficulties in interpreting MOC effects on DPOAEs and SFOAEs, some authors recommend measuring MOC effects at the output of the cochlea, e.g., by measuring the CAP (99). If OAEs must be used, it may be better to use click-evoked or transient-evoked OAEs and analyzing the results into frequency bands (100) rather than DPOAEs or SFOAEs. Marshall et al. (101), however, found a reasonably high within subject correlation between MOC effects assessed with SFOAEs and OAEs evoked by brief chirps. If DPOAEs must be used, it would be advisable to try to maximize the contribution from the distortion (f2) source by presenting a third tone with a frequency equal to 2f1 − f2 that suppresses the secondary (reflection) component (102). However, Marrufo-Pérez et al. (103) found no correlation between the effect of CAS on click-evoked OAEs analyzed into frequency bands and DPOAEs measured with a suppressor tone, which puts these recommendations into question.

In assessing MOC effects with OAEs or CAPs, the primary sounds should be short enough and the stimulation rate slow enough to prevent the probe stimuli from activating the MOC reflex by themselves. The human MOC response has an onset delay of between 25 and 40 ms and rise and decay constants in the region of 280 and 160 ms, respectively (reviewed in a later section). Therefore, a stimulation rate less than 30 per second would be appropriate. Similarly, when using psychoacoustical techniques, it would be advisable to use stimuli shorter than 25–40 ms to prevent them from activating the MOC reflex by themselves. This approach has been used to investigate ipsilateral MOC reflex effects of cochlear frequency selectivity (86) and on cochlear input/output curves (87). In addition, it is worth bearing in mind that the MOCR elicitor sound should start well before (>250 ms) the stimuli used to assess the MOC effects, or be continuous.

Finally, it would be important to control for the potential confounding effects of visual attention on MOC efferent effects (88) and to bear in mind that it may be unreasonable to assume identical characteristics for the ipsilateral and contralateral MOC reflex (17).

### Suppressive (Inhibitory) Effects in Silent Backgrounds

As noted earlier, in lower mammals, the activation of olivocochlear efferents suppresses cochlear responses to sounds in silent backgrounds. Many studies have confirmed a corresponding effect in humans using various methodologies. For example, CAS reduces the levels of sound-evoked (104) and spontaneous OAEs (105). The suppressive effect of CAS on evoked OAEs disappears after vestibular neurectomy (106), which supports the notion that it is mediated by olivocochlear efferents. The magnitude of OAE suppression varies across subjects, OAE modality, and CAS characteristics, reaching maximum values of 2–4 dB. The magnitude of suppression is greater for broadband than for narrowband CAS (98, 107) and increases with increasing the level of the CAS (104, 107–109). For a constant CAS level, the amount of suppression is greater for low than for high-level OAE probes, another characteristic consistent with the association of suppression of OAEs with active cochlear processes and efferent function at low-intensity levels (109).

The suppressive effect of olivocochlear efferent activation in silence has also been observed by comparing the CAP in the presence and in the absence of CAS and the magnitude of CAP suppression is typically larger than for OAEs (10 versus 2–4 dB) (99, 110, 111).

Some behavioral phenomena in humans are also consistent with the suppressive effect of olivocochlear efferents. For example, because auditory thresholds increase with decreasing the gain of the cochlear amplifier, and the activation of MOC efferents inhibits the amplifier gain, and MOC efferents may be activated by CAS, auditory thresholds should increase in the presence of CAS. This is indeed the case (112). The phenomenon has been referred to as “central masking” because the CAS was originally regarded as a “masker” and the threshold increase was interpreted to occur by interaction of that “masker” with the test tone somewhere in the central auditory nervous system (113). However, the current interpretation is that central masking is partly due to MOC inhibition of cochlear gain by the CAS (114).

Kawase et al. (115) found that (1) auditory thresholds for brief (50-ms duration) pure tones increased by >2–3 dB with broadband CAS at levels ≥30 dB SPL; (2) the threshold increase was larger for mid-frequency tones (2 kHz) than for tones with lower or higher frequencies; and (3) larger threshold increases with increasing the CAS level. Aguilar et al. (116) found an interaction between the increase in auditory threshold and the duration of the pure-tone probes. At 4 kHz, the threshold increase was larger for longer (500 ms) than for shorter (10 ms) probes, presumably because the detection thresholds were lower for the longer than for the shorter tones and MOC inhibition is greater at lower levels. At 0.5 kHz, by contrast, the increase was similar for long and short probes. They reasoned that MOC efferent activation inhibits human cochlear gain differently in the cochlear apex than in the base.

Some aspects in the published effects of CAS can appear somewhat inconsistent across studies. Some studies have reported greater effects at lower (0.5 kHz) than at higher (4 kHz) frequencies (82), while others have reported greater effects at mid frequencies (about 2 kHz) (115), and yet others have reported greater effects at higher (4 kHz) than at lower (0.5 kHz) frequencies [e.g., Ref. (116)]. One should bear in mind, however, that (1) the suppressive effects of CAS depend on the CAS bandwidth [e.g., Fig. 3 in Ref. (98)]; (2) OAE suppression decreases with increasing age in listeners with normal audiometry (117, 118); and (3) OAE suppression appears to be larger for OAE measured in the left than in the right ears (119), although other studies have reported greater suppression in the right ear (120). These factors (CAS bandwidth, age, and test ear) can differ across studies, which can make across-studies comparisons of CAS-activated MOC effects difficult. Nevertheless, some studies have reported little or no correlation between the effects of CAS on DPOAE, click-evoked OAEs, and increases in absolute thresholds measured in the same ear at the same probe frequencies (103) or decreases in cochlear mechanical gain (121), which suggests that CAS effects may reflect mechanisms other than MOC efferent suppression.

In summary, it is likely that CAS-evoked MOC activation suppresses human cochlear responses in silent backgrounds as it does in animals, but the characteristics of such suppression are still controversial possibly because the techniques used to activate the MOC efferents are indirect.

### Relative Strength of Ipsilateral, Contralateral, and Bilateral MOC Efferent Reflex in Humans

As noted earlier, in cats, about two-thirds of MOC fibers respond to ipsilateral sounds, nearly one-third respond to contralateral sounds and one-tenth respond to sounds presented to either ear (16). Furthermore, selective stimulation of MOC neurons with an electrode in the region of MOC cell bodies produces larger CAP changes in the contralateral than the ipsilateral ear (45). Based on this, one might think that, in humans, the ipsilateral MOC reflex should be “stronger” than the contralateral MOC reflex. However, the evidence in support of this assumption is inconclusive. Some studies have reported greater suppression of click-evoked (122) and transient (123) OAEs with ipsilateral than with contralateral broadband MOC elicitors. Other studies, by contrast, have reported greater suppression of SFOAEs with ipsilateral than with contralateral MOC elicitors only for half-octave wide sound elicitors; for two-octave wide or broadband elicitors, ipsilateral and contralateral elicitors produced similar amounts of suppression (98). In light of the latter, it seems too simplistic and likely erroneous to assume that the ipsilateral MOC reflex should be stronger than the contralateral reflex in humans.

Regarding bilateral MOC-elicitor sounds, some studies have reported their suppressive effects to be only slightly larger than for ipsilateral elicitors (122, 123). By contrast, Lilaonitkul and Guinan (98, 124) reported the suppression of SFOAEs caused by bilateral elicitors to be roughly equal to the sum of the suppression caused by ipsilateral and contralateral as measured separately, at least for broadband MOC elicitors.

### Time Course of Human Efferent Activation and Deactivation

Kim et al. (125) measured human DPOAE levels along the duration (5.5 s) of elicitor probes at 2, 4, and 5.7 kHz. DPOAE levels were maximal at the elicitor onset and decreased by as much as 3 dB over the elicitor duration. They modeled the time course of the decrease with two exponentials with time constants of 69 ms and 1.51 s. They referred to the decrease in DPOAE as “adaptation” and reasoned that it resembled the DPOAE adaptation found by Liberman et al. (67) in cats (reviewed above). Since the latter disappeared after sectioning the olivocochlear bundle, Kim et al. (125) reasoned that the adaptation of human DPOAEs was probably due to the DPOAE primary tones activating the ipsilateral MOC efferents, thus that the time constants of DPOAE adaptation probably reflected the time constants of ipsilateral MOC efferent activation.

A psychoacoustical study found that a precursor sound reduced the inferred gain of cochlear amplifier and that the recovery from inhibition could be described by an exponential with a time constant of 116 and 136 ms for precursors of 60 and 80 dB SPL, respectively (87). Assuming that the observed gain reduction was due to the precursor activating the ipsilateral MOC reflex, this indicated that the mean time course of deactivation of this reflex was about 126 ms.

James et al. (126) measured the suppression of DPOAEs by intermittent CAS and observed an onset delay in suppression that ranged 31–95 ms (mean 43 ms). On the assumption that the suppressive effects of CAS were due to activation of contralateral MOC efferents, this suggested that contralateral MOC efferents have a mean onset activation delay of about 43 ms.

Backus and Guinan (108) measured the suppression SFOAEs (evoked by 1 kHz probe tones) by ipsilateral, contralateral, and bilateral wideband noise. They found that suppression increased gradually after the noise onset as a saturating exponential with a time course of 277 ± 62 ms and decreased after the noise offset with a time constant of 159 ± 54 ms. For the “best” cochleae, however, the onset time course of suppression could be separated into “fast” (70 ms), “medium” (330 ms), and “slow” (25 s) components. In addition, they reported a 25 ms delay in onset and offset of suppression, thus broadly consistent with the findings of James et al. (126). Zhao and Dhar (127) found that CAS suppressed spontaneous OAEs by about 3.6 dB with a “fast” (3 s) and a “slow” (30 s) time constant, although most of the suppression occurred over the fast time period.

Konomi et al. (117) showed that the onset latency of DPOAE suppression by CAS increases with increasing age from about 60 ms for 2-year-old children to 150 ms for 50-year-old adults, without a change in the time constant of suppression. They argued that the increased latency might reflect deterioration in auditory brainstem function involved in the MOC reflex.

In summary, in humans as in animals, the time course of ipsilateral and contralateral olivocochlear efferent effects can be described as exponentially activating with several time constants that add up to about 300 ms, as exponentially deactivating with a time constant of about 160 ms, and with activation and deactivation delays of about 25–60 ms that can increase with increasing age.

### Frequency Tuning of Human MOC Effects

In response to pure tones, MOC fibers have narrow V-shaped tuning curves that are only slightly wider than the tuning curves of afferent fibers with similar CFs (16). This suggests that MOC efferents provide frequency-specific negative feedback on the cochlea to a narrow region around the sound frequency that activates the reflex. Several studies, however, suggest that this might not be the case in humans.

Lilaonitkul and Guinan (124) investigated the frequency tuning of the human MOC reflex at the 1-kHz cochlear region. To do it, they measured the effect of ipsilateral, contralateral, and bilateral MOC-elicitor frequency on the suppression of SFOAEs. They found that for ipsilateral tonal and narrowband elicitors, the largest MOC effects were from elicitors centered at the SFOAE probe frequency (1 kHz), as expected. For contralateral and bilateral elicitors, by contrast, the largest effects were for elicitors about half an octave below the SFOAE probe frequency. This conclusion, however, does not apply to other probe frequencies. Indeed, the same authors have reported that MOC elicitors with frequencies in the range 0.5–2 kHz were particularly effective in suppressing SFOAEs at probe frequencies near 0.5 and 1 kHz, regardless of elicitor laterality. At 4 kHz, however, the most effective elicitor frequency was 4 kHz for ipsilateral and bilateral elicitors, and 0.5–4 kHz for contralateral elicitors (128). Similarly, Zhao and Dhar (129) reported that contralateral MOC elicitors were most effective in suppressing the level of spontaneous OAEs when the elicitor frequency was between 0.5 and 1 kHz, regardless of the frequency of the spontaneous OAE. A behavioral study has reported that cochlear gain at 4 kHz was reduced by ipsilateral MOC elicitors with frequencies up to 0.5 octaves below and above the probe frequency (130). Altogether, the existing evidence suggests that the most effective MOC-elicitor sounds have frequencies in the range 0.5–2 kHz, regardless of elicitor laterality.

A related question is what is the most effective MOC-elicitor bandwidth? Lilaonitkul and Guinan (98) reported that the magnitude of SFOAE suppression increased asymptotically with increasing the MOC-elicitor bandwidth. Maximal suppression occurred for elicitor bandwidths ≥4 octaves relative to the SFOAE probe frequency. The effect of elicitor bandwidth was similar for SFOAE probe frequencies of 0.5, 1, or 4 kHz, and for ipsilateral, contralateral, and bilateral elicitors.

### Changes in Human Cochlear Gain and Compression

**Figure 4:**
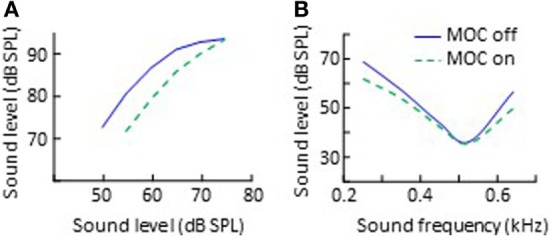
Effects of CAS-evoked medial-olivocochlear (MOC) activation on human behaviorally inferred cochlear input/output curves **(A)** and psychoacoustical tuning curves **(B)**. The data in panel **(A)** are re-plotted, in modified form, from Fig. 2 in Krull and Strickland (83), subject S1. The data in panel **(B)** are re-plotted, in modified form, from Fig. 3 in Aguilar et al. (82).

As reviewed earlier, animal studies have shown that the primary effect of MOC activation is to reduce the gain of basilar membrane responses to low-level sounds, thus to linearize cochlear mechanical input/output curves (Figure 2A). The same seems to occur in humans. Cochlear input/output curves inferred using behavioral methods have 2–20 dB less gain when measured with precursor sounds [e.g., Ref. (81, 83, 87)] or with CAS (84, 121) designed to activate the ipsilateral and the contralateral MOC reflex, respectively (Figure 4A). The gain reduction is larger the higher the level of the MOC-elicitor sound (87).

Most gain-reduction studies have investigated MOC effects at frequencies 2 kHz or higher. Aguilar (84), however, reported larger gain reductions at 500 Hz than at 4 kHz with CAS-evoked MOC activity. This is broadly consistent with the greater reduction of OAE levels at frequencies around 0.5 and 1 kHz than at higher frequencies [e.g., Ref. (98)], but inconsistent with the notion that the MOC-induced inhibition of gain should be greater in the base than in the apex because cochlear gain is greater in the base than in the apex (40). In addition, Aguilar (84) showed that although CAS inhibited responses to low-level sounds, it sometimes increases responses to moderate or high-level stimuli, which is broadly consistent with physiological findings in rodents (33, 74).

### Changes in Human Cochlear Frequency Selectivity

Animal studies have also shown that MOC efferent activation reduces the amplitude of cochlear mechanical responses for stimulus frequencies near the CF more than for frequencies remote from the CF, thereby broadening cochlear mechanical tuning curves, shifting their tips upwards [cf. Fig. 2 in Ref. (33)]. Several studies suggest that the same occurs in humans. For example, CAS-evoked MOC activity reduces the time delay at which transient OAEs reach their peak level [e.g., Ref. (100, 131)]. According to linear filter theory, the shorter the time delay of a filter’s impulse response, the broader the filter is. Transient OAEs reflect the impulse response of cochlear filters. Therefore, the shorter OAE delays with, than without CAS-evoked MOC activity indicate that MOC activity broadens cochlear frequency responses.

Medial-olivocochlear activation also broadens auditory frequency selectivity. Psychoacoustical frequency tuning curves (a behavioral correlate of cochlear tuning curves) are generally broader with, than without MOC activity evoked by CAS (Figure 4B) (82, 132) or by ipsilateral precursor sounds (81, 133). Binaural auditory filters are also broader with MOC activity evoked by a precursor sound (134). Interestingly, for some procedures, the effect of CAS is to shift the tails of the psychoacoustical tuning curves downward rather than their tips upward (compare Figure 4B with Figure 2B). A detailed explanation for the different effect of CAS on physiological and psychoacoustical tuning curves is out of the scope of this review but can be found elsewhere (82).

The majority of studies report the broadening effect of sound-evoked MOC activity to be small (about 5%) and for it to be larger at frequencies below than above 2 kHz (82, 100). Some studies, however, have reported that CAS-evoked MOC activity broadened cochlear tuning at 2 and 4 kHz but sharpened it at 0.5 or 1 kHz (119, 135).

### Antimasking Effects in Noisy Backgrounds

As noted earlier, animal studies have shown that the activation of the olivocochlear efferents can enhance neural responses to brief sounds in low-level noise (Figure 3A). The evidence for a corresponding “antimasking” effect in humans is very controversial. Scharf et al. (93) showed that vestibular neurectomy does not affect the thresholds for detecting pure tones presented to one ear embedded in ipsilateral or in binaural (dichotic) noise, which suggests that olivocochlear efferents neither facilitate nor degrade the detection of tones in noise. In contrast with this, Micheyl and Collett (136) showed that listeners who had greater suppression of OAE levels by broadband CAS had better thresholds for detecting 2 kHz pure tones embedded in broadband binaural (dichotic) noise. Assuming that the stronger OAE suppression reflected a stronger contralateral MOC reflex, this suggested that the stronger the MOC reflex, the greater the antimasking effect of the CAS. One possible explanation for the discrepancy between the two studies is, perhaps, that vestibular neurectomy does not cut the olivocochlear efferents (95). However, Verschooten et al. (137) did not find greater CAP responses to masked tones in the presence of a precursor sound that was expected to activate the ipsilateral MOC efferent reflex and produce “antimasking.”

A perceptual phenomenon that has been related to the antimasking effects of olivocochlear efferent activation is the so-called “overshoot.” Overshoot refers to the improved detectability of a probe tone embedded in noise when the tone is delayed from the noise onset or when it is preceded by a precursor sound (138). While overshoot was first explained in terms of short-term adaptation in the auditory nerve, some characteristics of overshoot are not consistent with this explanation [reviewed in Ref. (139)]. Many authors have suggested that overshoot is caused by a reduction of cochlear mechanical gain mediated by the MOC efferents (133, 140–146). The leading noise or the precursor sound would activate the ipsilateral MOC reflex, thus reducing the cochlear mechanical gain, which would reduce the amount of masking [for detailed explanations, see Ref. (143, 144)]. The most recent studies, however, provide compelling evidence that overshoot is unrelated with an MOC-related reduction in cochlear gain and undermine the link between overshoot and MOC efferents [(137, 147, 148); see also Ref. (149)].

In summary, the evidence for an antimasking effect of olivocochlear efferents in humans is not compelling.

## Roles of the Olivocochlear Efferent Reflexes in Human Hearing

This section addresses a long standing question: what role(s) do olivocochlear efferents play in human hearing?

Walsh et al. (150) showed that, in neonatal cats, de-efferentation elevates the discharge-rate threshold and broadens the frequency tuning curves of afferent auditory nerve fibers. These characteristics are typical of auditory nerve fiber responses in cochleae with OHC loss or dysfunction (i.e., they are typical of a damaged cochlear “amplifier”) but in the de-efferented cochleae of neonatal cats they occurred in the absence of obvious histological OHC damage. This suggests that efferents may be essential for normal development of cochlear active mechanical processes.

Other authors have proposed that efferents may protect the auditory system from excessive acoustic stimulation. Cody and Johnstone (151) showed that, in guinea pigs, CAS reduced the temporary loss of auditory sensitivity caused by intense sounds. They reasoned that the CAS activates the MOC efferents, which inhibit cochlear mechanical responses to the intense sound, and thus reduce the desensitizing effect of this sound. Olivocochlear efferents may also decrease the risk of permanent noise-induced [(152); see also Ref. (72)] and age-related (153) hearing loss. In addition, efferents may protect from noised-induced cochlear neuropathy (154), a permanent subclinical condition (155) that could underlie the greater difficulty understanding speech in noisy backgrounds experienced by some people with clinically normal hearing (156, 157). However, while the evidence for a protective effect of olivocochlear efferents is strong in animals, the evidence for a corresponding role in humans is equivocal [reviewed in Ref. (158); see also Ref. (159)].

The “antimasking” effect of efferent activation on neural physiological responses have led to the notion that efferents facilitate the detection of signals in noise. In particular, efferents may facilitate the detection and recognition of speech in noise. Although deeply investigated, the evidence in support of this notion is still controversial. For example, speech-in-noise recognition is worse in some but not all vestibular neurectomy subjects (94, 160). Because neurectomy does not affect all subjects equally and is ineffective in cutting the olivocochlear efferents (95), this undermines the conclusion that the worse speech-in-noise recognition of the affected neurectomy subjects is due to their lacking an olivocochlear efferent system. In addition, some studies have reported better speech-in-noise recognition for subjects with stronger MOC suppressive effects [e.g., Ref. (120, 161)], while others have found the opposite trend [e.g., Ref. (162, 163)]. Bidelman and Bhagat (120) reported that the hypothesized correlation between olivocochlear efferent suppression of OAE levels and speech-in-noise recognition occurs for the right ear but not for the left ear. Mertes et al. (164) have reported that efferent suppression of OAE levels correlates with the slope of the psychometric function for speech recognition score as a function of signal-to-noise ratio but not with any single point on that function.

The cortex exercises control over the function of the cochlea [(165, 166); reviewed in Ref. (26)], even with anesthesia [e.g., Ref. (167)]. Both auditory and visual selective attention modulates cochlear responses via olivocochlear efferents [e.g., Ref. (88, 168–173)]. Because of this, and given the controversial evidence for an antimasking effect of the olivocochlear reflex, de Boer et al. (162) proposed that “the MOC system benefits speech-in-noise processing through dynamic (e.g., attention dependent and experienced dependent), rather than reflexive control of cochlear gain.” Although possible, this view clashes with evidence that signal processing strategies designed to reinstate reflexive olivocochlear efferent effects improve auditory perception by hearing-aid users (174) as well as the recognition of speech in noise by cochlear implant users (175, 176). It also clashes with the fact that artificial speech recognizers operating on neural rather than acoustic representations of speech perform better in noisy backgrounds when the neural representation encompasses reflexive olivocochlear efferent effects (177, 178).

The reasons for the disparity across studies remain uncertain. Perhaps, it is due to the use of a single value, such as the speech reception threshold (SRT), for quantifying speech recognition in noise and/or to the lack of experimental control of factors such as laterality or selective attention (179).

Other authors have proposed that olivocochlear efferents facilitate the spatial localization of sound sources in noise, but the evidence in support of this is somewhat mixed. Listeners who show larger suppression of OAE levels by CAS-evoked MOC activity tend to be more accurate at localizing sounds sources in the vertical plane (180) but not in the horizontal plane (181). Irving et al. (182) showed, in ferrets, that olivocochlear efferents are unnecessary for accurate sound localization in the horizontal plane but are involved in re-learning new localization cues after unilateral hearing loss. de Boer and Thornton (183) gave evidence that in humans, MOC activity increased with auditory learning, and that the increase was larger for those listeners who improved significantly in the auditory task.

In summary, olivocochlear efferents may play multiple roles in human hearing. The evidence in support of each one of those roles remains, however, controversial. Smith and Keil (184) reasoned that the fact that olivocochlear efferents can play multiple roles in hearing does not mean that they evolved naturally to play all those roles. They reasoned that ecological acoustic environments hardly ever contain very intense sounds. Therefore, it is unlikely that olivocochlear efferents evolved to protect the auditory system from acoustic trauma. Instead, they probably evolved to facilitate the detection of signals in noise by inhibiting the cochlear mechanical gain for the unattended sounds.

### How Much Do Efferents Help Understanding Speech in Noise?

As noted earlier, in laboratory tests designed to maximize the magnitude of efferent effects, MOC efferent activation causes at most about 10–15 dB reduction in basilar membrane sensitivity (Figure 2A) and about 5 dB increase in auditory sensitivity in noise as measured by CAP (Figure 3A). These effects are small in magnitude and are likely to be even smaller during natural listening to ecological sounds. Therefore, one might wonder: how much can efferents facilitate the recognition of speech in noisy environments?

This challenging question has been addressed in several different ways. Zeng et al. (160) compared SRTs (defined as the signal-to-noise ratio at 50% recognition) in diotic noise for the two ears of unilaterally neurectomized listeners. They found SRTs to be between 2 and 10 dB worse (higher) for the surgery than for the non-surgery ear. Unfortunately, the subjects tested by Zeng et al. had hearing loss in the surgery ear, which could have led to the higher SRTs in the surgery ear. Giraud et al. (94) reported a similar experiment with dichotic noise in unilaterally neurectomized listeners who had normal audiometric thresholds in both ears. They found that phoneme recognition in the surgery ear did not improve with CAS while it improved between 12 and 24% in the non-surgery ear.

Brown et al. (177) addressed the same question using an automatic speech recognizer that operated on computational model simulations of auditory nerve responses to speech rather than on the acoustic speech. They found that for speech tokens presented at 60 dB SPL in competition with noise at 50 dB SPL, the recognizer went from recognizing 10% of the speech tokens without efferent attenuation to recognizing 50% of the speech tokens with 10 dB MOC attenuation of basilar membrane gain (their Fig. 6). They further showed that the improvements in speech recognition were larger for lower noise levels (their Fig. 7). Lopez-Poveda et al. (175, 176) reported that, for users of cochlear implants, SRTs in steady-state noise or single-talker interferers improved by up to 7 dB when using sound processors that involved contralateral gain inhibition inspired by the MOC efferent reflex compared with using conventional processors without simulated efferent control.

Although the cited studies employed indirect methods that may not be accurate in revealing MOC efferent benefits for listeners with normal hearing, their results indicate that, despite causing small changes in cochlear responses to sounds, MOC efferents can significantly improve the recognition of speech in noise.

## Clinical Relevance and Special Populations

Several studies have demonstrated an association between abnormal olivocochlear efferent function and various health conditions. For example, reduced MOC reflex strength has been associated with auditory processing disorders [e.g., Ref. (185, 186); reviewed in Ref. (187)], dyslexia [e.g., Ref. (188)], ankylosing spondylitis (189), migraine and phonophobia in women (190), or poorer speech-in-noise recognition and language impairment in children (191). Efferent suppression of cochlear activity by the MOC efferent system appears to be enhanced in individuals with tinnitus and/or hyperacusis (192, 193). One study has given evidence that the hypersensitivity of autistic children to sounds is associated with a weaker olivocochlear efferent system (194), while other study reports the opposite trend; i.e., hyperacusis in autistic children is correlated with stronger MOC efferent suppression (195). Some epileptic hamsters show morphofunctional alterations of the olivocochlear efferent system, which might contribute to the susceptibility of these hamsters to audiogenic seizures (196). The poorer speech-in-noise intelligibility of cochlear implant users may be partly due to these users lacking the “antimasking” effects of the MOC reflex (175, 176). Intensive listening experience(s), such as for example musicianship, can strengthen the ipsilateral and contralateral MOC efferent system and sound regulation to the inner ear, which presumably reduces acoustic vulnerability to damaging sounds (197).

## Open Issues and Outlook

As reviewed, many aspects about the anatomy, physiology, and function of olivocochlear efferents have been settled over the last few decades. Many other aspects, however, remain open. For example, if MOC efferent activation suppresses the gain of the cochlear amplifier, why and how does it sometimes change cochlear mechanical responses for stimulus frequencies presumably unaffected by the cochlear amplifier (i.e., stimulus with frequencies remote from the CF)? If MOC efferent activation typically suppresses responses to sounds in silent backgrounds, why and how does it sometimes *enhance*, rather than suppress, basilar membrane responses? Why is the frequency of the most effective MOC-elicitor sound typically about 0.5–2.0 kHz, regardless of the probe frequency used to asses MOC effects? How do these aspects affect hearing?

Also open are many issues regarding human olivocochlear efferents. For example, what is the most accurate and reliable method for assessing MOC effects in humans? Can a method be developed to separate out the simultaneous effects of MOC and LOC efferents on human neural responses to sound? What explains the low (or lack of) within subject correlation of MOC effects assessed using different (but carefully designed) methods? To what extent does uncontrolled attention affect olivocochlear efferent effects assessed with techniques that do not demand attention (e.g., OAEs)? Do human olivocochlear efferents really facilitate the detection of sounds in noisy backgrounds? If so, under what conditions? To what extent are olivocochlear efferent effects affected by the various types of hearing impairment? Can a method be developed to monitor olivocochlear efferent effects *during* natural listening? What role(s) do olivocochlear efferents play in human hearing? Do they function differently during active and passive listening? Are efferents actually involved in learning how to listen? Could they be facilitating the transmission of information of interest (the attended sounds) by suppressing the irrelevant information (the unattended sounds)?

Further research is necessary to address these and other open questions. To this end, cochlear implants offer a novel and potentially useful approach. Cochlear implants restore hearing to some deaf individuals by direct electrical stimulation of the auditory nerve (198); that is, they effectively function as “artificial ears.” The electrical stimulation provided by a cochlear implant bypasses the OHCs, the site of action of MOC efferents, and is independent from MOC effects. As a result, the users of cochlear implants lack MOC efferent effects but may have the effects of LOC efferents. Therefore, insights into the roles of olivocochlear efferents in hearing may be gained by comparing auditory performance by normal-hearing individuals with that of cochlear implant users (199). In addition, cochlear implants allow unique control over the electrical stimulation used to evoke auditory sensations. Stimulation strategies have been designed for cochlear implants that roughly mimic the effects of MOC efferent activation [e.g., Ref. (175, 200)]. Unlike natural efferents, the MOC effects mimicked with these strategies can be turned on and off at will, which allow within subject comparisons of auditory performance with and without MOC efferent effects. In summary, cochlear implants offer interesting possibilities to address some of the open questions regarding the roles of olivocochlear efferents in hearing.

## Author Contributions

EAL-P did the research and wrote the paper.

## Conflict of Interest Statement

The author declares that the research was conducted in the absence of any commercial or financial relationship that could be construed as a potential conflict of interest.
